# The High Solid Loading and Stability of SiO_2_ Ceramic Slurry for Stereolithography

**DOI:** 10.3390/ma19102071

**Published:** 2026-05-15

**Authors:** Wenlu Zhang, Chunfa Huang, Shengjun Xia, Xing Hu, Qiulin Li

**Affiliations:** 1 Institute of Materials Research, Shenzhen International Graduate School, Tsinghua University, Shenzhen 518055, China; 2 School of Materials Science and Engineering, Tsinghua University, Beijing 100084, China

**Keywords:** silica, stereolithography, additive manufacturing, high solid loading, stability

## Abstract

Stereolithography (SL) based additive manufacturing technology facilitates fabricating structurally complicated silicon-based ceramic cores. However, SL fabricates ceramic component by layer-by-layer solidification of ceramic slurries. The instability of ceramic slurries often leads to the phenomenon of ceramic particle gradient distribution in the prepared ceramic component, which can damage the mechanical properties, porosity, and dimensional accuracy of ceramic component, hindering the application of SL in the preparation of ceramic component. Herein, we systematically investigated and optimized such impacting factors, including particle size distributions, addictive (nanopowder) and solid loading, which result in appropriate viscosity and high stability of SiO_2_ ceramic slurries. A SiO_2_ ceramic slurry for SL showed the viscosity (26.1 Pa·s) and after 264 h sedimentation time with 80 wt.% of solid loading. Using the slurry, we prepared a ceramic with a shrinkage rate maintained below 4%, a porosity of 21.56%, and a flexural strength of 20.53 MPa.

## 1. Introduction

Ceramic cores are essential components for the fabrication of turbine blades in aerospace engines, constructing complex internal cooling channels within the engine’s blades [1,2,3]. As the performance of aerospace vehicles continues to improve, there is an increasing demand for higher temperature capabilities in turbine engines, leading to more intricate internal structures of the turbine blades [4,5,6]. Additive manufacturing has overcome the limitations of traditional investment casting, which are constrained by mold design and long preparation cycles [7]. Additive manufacturing technologies for fabricating ceramic cores include selective laser sintering (SLS) [8,9], direct ink writing (DIW) [10,11], and stereolithography (SL) [5,12]. Ceramic cores produced by stereolithography exhibit high dimensional accuracy, moderate porosity, and a certain level of flexural strength, making it a widely utilized method for ceramic core manufacturing [4,13,14,15,16].

Comparable to the traditional investment casting used for ceramic core production, additive manufacturing techniques also utilize refractory materials such as SiO_2_ [17,18,19,20], Al_2_O_3_ [21,22,23,24], and ZrO_2_ [4,11,20,25,26,27]. In contrast to conventional methods, stereolithography additive manufacturing involves the solidification of ceramic slurries through exposure to specific wavelengths of light, which allows for the creation of the desired component [28]. The propagation of light through the slurry determines the dimensional accuracy of the cured ceramic core [29]. Compared to Al_2_O_3_ and ZrO_2_, SiO_2_ has a smaller refractive index (RI) difference with commonly used photopolymerization resin systems, resulting in a larger curing depth and smaller mis-curing dimensions under the same light intensity [30,31,32,33]. Therefore, SiO_2_ demonstrates superior dimensional accuracy. Furthermore, SiO_2_ offers the benefits of low shrinkage and ease of dissolution in alkaline solutions post-casting, which are pivotal to its widespread adoption as the preferred material for ceramic core fabrication [34,35,36,37].

**Figure 1:**
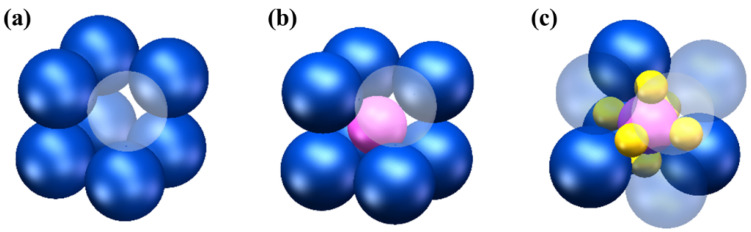
Schematic diagram of particle accumulation: (**a**) monomodal particle size distribution; (**b**) bimodal particle size distribution; (**c**) trimodal particle size distribution.

The dimensions of turbine blades and ceramic cores are increasingly trending towards larger sizes [38]. In the laboratory setting, there is less focus on the stability of ceramic slurries, possibly due to the common practice of fabricating small samples and test bars at the centimeter scale, which can be rapidly cured without the prolonged printing time that might lead to gradient distributions of material, and cause significant performance issues. However, in the actual fabrication of large parts, a longer curing time is required for layer-by-layer curing, necessitating a slurry with a certain level of stability and uniform dispersion to prevent gradient effects and property degradation [6]. Furthermore, commercial slurries, which are subject to various processes including preparation, encapsulation, transportation, and storage, must exhibit high stability to ensure consistency and performance throughout these stages. Guojiao Ding studied the effects of resin monomers, dispersants, and ceramic particle sizes on the stability of SiC slurries after 60 h of settlement and found that a 1:1 mass ratio of HDDA to TMPTA resin monomers could enhance slurry stability [39]. Keqiang Zhang investigated the impact of dispersant types and concentrations on the stability of Al_2_O_3_, discovering that when KOS110 was used as the dispersant at a concentration of 5 wt.%, the slurry remained uniformly stable for up to 240 h [40]. Regrettably, previous research has mainly concentrated on Al_2_O_3_, SiC and ZrO_2_ [41,42,43], with less systematic study on the stability of the widely used SiO_2_ ceramic slurries. Furthermore, after a period of sedimentation, although the slurry may not show obvious sedimentation macroscopically, different particle sizes within the slurry may settle differently due to varying forces acting upon them, potentially leading to disparities in the micro distribution of particles within the slurry. The ceramic powder forms the skeleton of the ceramic core and is one of the critical factors affecting its properties. Variations in particle size distribution within the slurry lead to distinct packing arrangements, as shown in Figure 1, where distinct packing arrangements result in varying numbers of particles per unit volume. Nanopowder, due to their diminutive size, are capable of occupying finer interstices within a matrix. The particle concentration per unit volume of the slurry varies with different solid loadings, which in turn can affect the settling behavior of particles and the viscosity of the slurry. Consequently, it is essential to systematically investigate the synergistic effects of particle-size distribution, nanopowder addition, and solid loading on the rheological behavior and prolonged stability of ceramic slurries. Achieving a balance among high solid loading, suitable printability, and prolonged suspension stability remains a key challenge in stereolithography-based ceramic processing.

In addition to ceramic core fabrication, SiO_2_-based materials have also attracted considerable interest in advanced optical, electronic, and energy-related applications due to their excellent thermal stability, chemical resistance, and dielectric properties. Recent studies have demonstrated the potential applications of SiO_2_ materials in solar-energy-related systems, plasma-processing technologies, and electromagnetic interference (EMI) shielding materials [44,45,46]. Therefore, the development of highly stable and printable SiO_2_ ceramic slurries is also of potential significance for the fabrication of complex SiO_2_-based components in advanced functional applications.

Hence, this study systematically investigates the effects of multimodal particle-size distribution, nanopowder addition, and solid loading on the stability and rheological behavior of SiO_2_ ceramic slurries. In particular, this work aims to clarify the particle-configuration principles governing slurry stability and printability, and to establish an effective strategy for designing highly stable SiO_2_ slurries suitable for stereolithography processing.

## 2. Materials and Methods

### 2.1. Raw Materials

In order to study the effect of particle size distributions, SiO_2_ spherical ceramic powders with D50 of 20 nm, 1 μm, 4 μm, 8 μm and 23 μm were selected as raw materials with purity above 99.7%. Ceramic powders were purchased from Zhejiang Manli Nano Technology Co., Ltd., Yuyao, Zhejiang, China.

In this study, two resin monomers, namely 1,6-hexanediol diacrylate (HDDA) and trimethylolpropane triacrylate (TMPTA), were used to prepare the photocurable resin system, with a mass ratio of 1:1. The photoinitiator diphenyl (2,4,6-trimethylbenzoyl) phosphine oxide (TPO, purity 90%) was added at 2 wt.% relative to the total mass of the resin system. The remaining resin mass consisted of HDDA and TMPTA monomers. HDDA, TMPTA, and TPO were purchased from RYOJI, Guangzhou, Guangdong, China. Triton X-100 was used as the dispersant at an addition amount of 1 wt.% relative to the total mass of the ceramic slurry and was purchased from Dow Chemical Company, Midland, MI, USA.

### 2.2. Slurry Preparation

To prepare the SiO_2_ slurry, followed these steps: Accurately weighed the SiO_2_ powder, resin monomer, photoinitiator, and dispersant, and transferred them into a 500 mL stirring tank. Added 6 ZrO_2_ grinding balls with a diameter of 12 mm to the tank, placed the tank into the planetary ball mill, and set the grinding speed to 600 rpm. Mixed for a duration of 40 min to achieve thorough blending of the ingredients.

### 2.3. Stereolithography and Debinding Sintering

Utilizing a commercial Digital Light Processing (DLP) additive manufacturing equipment (Adventure-3D-WH-Printer-96-50, Adventure Technology, Shenzhen, Guangdong, China), the SiO_2_ ceramic slurry was cured using light polymerization. The cured layer thickness was set at 250 μm, with an irradiance of 8.89 mW/cm^2^, a curing duration of 2 s, and a UV wavelength of 405 nm. The slurry was poured into a reservoir, and the build platform moved layer-by-layer with the assistance of a blade for sequential curing, resulting in the fabrication of SiO_2_ ceramic green bodies.

Subsequently, the green bodies were subjected to a debinding and sintering process in a muffle furnace (KSL-1700X-A2, Kejing, Hefei, Anhui, China). The temperature was increased from room temperature to 100 °C at a heating rate of 1 °C/min. From 100 °C to 600 °C, the heating rate was reduced to 0.5 °C/min to ensure the gradual release of gases generated during resin oxidation and decomposition, thereby reducing crack formation in the green bodies. From 600 °C to the final sintering temperature, the heating rate was increased to 3 °C/min. In addition, the samples were held for 2 h at 100 °C, 200 °C, 300 °C, 420 °C, 500 °C, 600 °C, and 800 °C, respectively. Finally, the samples were sintered at 1300 °C for 6 h and then furnace-cooled to room temperature.

### 2.4. Characterization

The particle size distribution of SiO_2_ particles was determined using a laser particle size analyzer (LA-950V2, HORIBA, Kyoto, Japan), and the microscopic morphology was observed with a scanning electron microscope (MIRA LMS, TESCAN, Brno, Czech Republic). The viscosity of the SiO_2_ slurry was tested at room temperature using a rotary viscometer (DV2T, Brookfield, Middleboro, MA, USA). For each slurry, the viscosity measurement was repeated five times to ensure the reliability and reproducibility of the experimental results. The viscosity values reported in this study correspond to measurements obtained at a rotational speed of 6 rpm. The rheological properties of the ceramic slurry was tested at different shear rates using a rheometer (MCR302e, Anton Paar, Graz, Austria) at room temperature. The stability of the SiO_2_ slurry was measured through a static sedimentation experiment, where the slurry was loaded into a 15 mL test tube with a graduated scale. After standing for a specific period, the ratio of the volume of the supernatant (V0 − V1) to the initial volume of the slurry (V0) is defined as the sedimentation coefficient of the slurry, thereby providing a measure of the slurry’s stability. Based on the sedimentation coefficient equation, the degree of separation between the particle-rich phase and the liquid phase after sedimentation can be quantitatively evaluated. A lower sedimentation coefficient indicates that particle sedimentation is more effectively suppressed and that particles remain more uniformly dispersed within the slurry, corresponding to better suspension stability. The sedimentation coefficient is influenced by multiple factors, including slurry viscosity, particle size, particle-size distribution, and interparticle interactions, and therefore can reflect the overall stability behavior of ceramic suspensions during storage and stereolithography processing.

The porosity and bulk density of the sintered body were determined using the Archimedes’ method. The measurements were conducted using a ceramic density meter (MAYB303C, Mayzum, Shenzhen, Guangdong, China). In addition, shrinkage and mechanical property measurements were performed to verify the feasibility of fabricating ceramic components using the optimized slurry system. The mechanical properties of the sintered body were evaluated using a universal testing machine (LE3104, Lishi Instrument, Qinhuangdao, Hebei, China) employing the three-point bending test. The dimensions of the sintered body were 40 mm × 10 mm × 4 mm.

## 3. Results and Discussion

### 3.1. Effects of Particle Size Distribution

Spherical SiO_2_ ceramic powders with sizes of 23 μm, 8 μm, 4 μm, and 1 μm were used to create monomodal, bimodal, and trimodal particle size distributions. Among these, the trimodal particle size distributions employed various gradations. Slurries with a solid loading of 60 wt.% were prepared and the viscosity of each slurry was measured. Then the slurries were subjected to a 264 h sedimentation experiment. For simplicity, slurries with monomodal, bimodal, trimodal particle size distributions are correspondingly named as M, B, and T, respectively. The particle size distributions information of the SiO_2_ slurries in each tube is listed in Supplementary Table S1 of Supplementary Materials.

**Figure 2:**
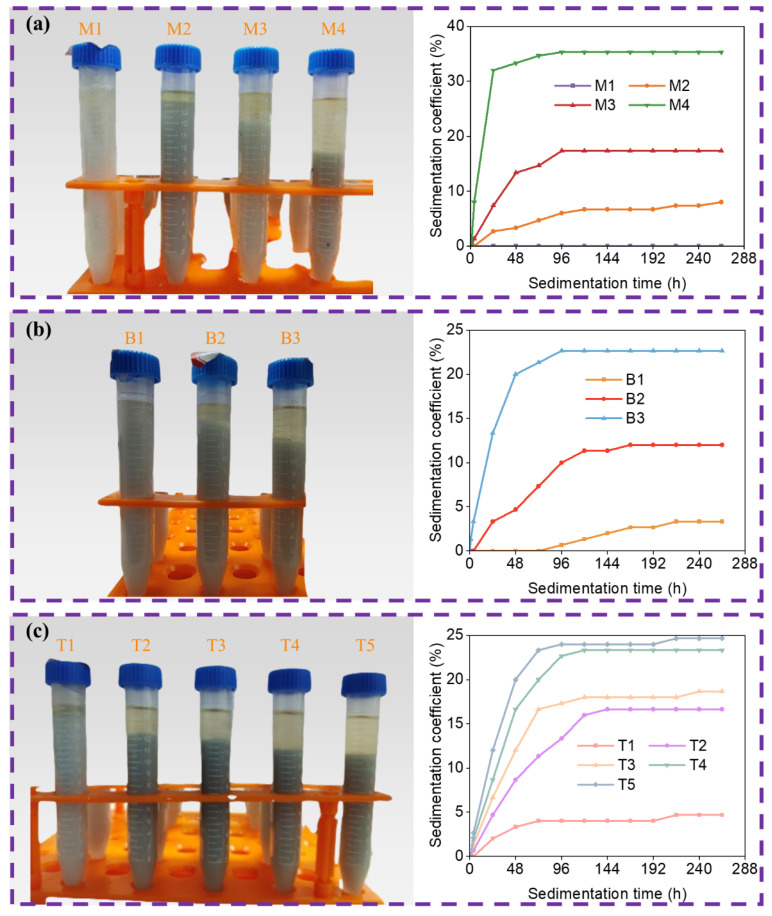
The delamination condition and sedimentation coefficient of slurry with time after standing for 264 h: (**a**) Monomodal particle size distributions; (**b**) bimodal particle size distributions; (**c**) trimodal particle size distributions.

After a 264 h sedimentation period, the layering of slurries with various particle size distributions, as well as the evolution of the sedimentation coefficient over time, are illustrated in Figure 2. Based on the changes in sedimentation coefficient with time, the sedimentation process can be divided into two distinct stages in terms of time. In the first stage, as time progresses, the sedimentation coefficient increases, referred to as the sedimentation period. During this period, the rate of change in the sedimentation coefficient initially accelerates and then decelerates, thus dividing the sedimentation period into a rapid sedimentation period and a slow sedimentation period, or a pre-sedimentation period and a post-sedimentation period. The sedimentation movement primarily occurs in the early sedimentation period of the sedimentation period. In the second stage, as time progresses, the sedimentation coefficient remains relatively constant, referred to as the stable period. It is evident that when the particle size is small, there is no significant sedimentation occurs, meaning that there is only a stable period, without a sedimentation period. However, when the particle size reaches a certain threshold, the sedimentation period begins to emerge. With the further increase of particle size, the duration of the pre-sedimentation period shortens, while the duration of the post-sedimentation period extends. During the pre-sedimentation period, particles undergo most of their sedimentation journey in a short time frame, with larger particles sedimenting more rapidly than smaller ones.

The sedimentation behavior of ceramic particles within a slurry is often discussed with the aid of Stokes’ formula. The settling velocity of ceramic particles in the slurry can be calculated using Stokes’ formula [47]:

$$v=\frac{\left(\rho_{powder}-\rho_{liquid}\right)gd_{powder}^{2}}{18\mu_{liquid}}$$

where *ρ_powder_* is density of the ceramic particle, *ρ_liquid_* is the density of the fluid, g is the acceleration due to gravity, *d_powder_* is the diameter of the ceramic particle, μ is the dynamic viscosity of the fluid. It should be noted that the classical Stokes’ law mainly describes the sedimentation behavior of isolated particles in dilute Newtonian systems and cannot fully account for the complex interparticle interactions, hindered sedimentation, and non-Newtonian rheological behavior in highly concentrated ceramic suspensions. Therefore, in the present study, Stokes’ law is primarily used to qualitatively explain the general sedimentation tendency of particles with different particle sizes.

According to Stokes’ formula, the settling velocity of particles in the slurry is related to the density of the ceramic particles and the resin system, the radius of the ceramic particles, and the dynamic viscosity of the resin system. In the pre-sedimentation period, the ceramic particles are uniformly distributed in the ceramic slurry, with the slurry having a relatively low viscosity, leading to rapid particle settling. As the ceramic particles continue to settle, the upper layer of the slurry has fewer ceramic particles, resulting in a slight decrease in viscosity. Conversely, the lower layer, with an increased number of ceramic particles, experiences an increase in viscosity, further impeding the settling velocity of the slurry. This leads to a decrease in the settling velocity of the ceramic particles and a flattening of the time-dependent change in the sedimentation coefficient, marking the transition into the post-sedimentation period. With further particle settling, the lower layer of the test tube accumulates an even greater number of ceramic particles, reducing the inter-particle spacing. Due to the interparticle interactions, such as van der Waals forces, and the interaction between the particles and the resin system, the particles can only move within a limited range, resulting in a macroscopic cessation of sedimentation. This aligns with the prediction of Stokes’ formula, which describes the settling velocity of particles in the slurry decreasing as the particles settle, transitioning from a lower viscosity in the upper layer to a higher viscosity in the lower layer.

In stereolithography, the stability of the ceramic slurry is paramount to the printing process and the quality of the final product. The particle size distributions significantly influences the settling behavior of the slurry. Figure 2 illustrates that as particle size increases, the sedimentation coefficient of the slurry also rises, indicating a more pronounced phase-separation behavior and poorer suspension stability.

This trend is particularly pronounced in the trimodal particle size distributions, where an increase in the proportion of larger particles leads to a significant increase in the sedimentation coefficient. However, the introduction of a greater number of particle size distributions is observed to decrease the sedimentation coefficient, suggesting that a multi-modal particle size distribution can enhance the stability of the slurry. The even dispersion of smaller particles throughout the slurry plays a crucial role in maintaining stability by effectively preventing the sedimentation of larger particles.

In the green body of ceramic cores, large particles serve as a skeleton, which not only increases the solid loading and porosity of the slurry but also enhances the strength of the ceramic cores [12,48]. Concurrently, smaller particles, due to their easier fusibility, play a binding role during sintering, further reinforcing the strength of the ceramic cores [49]. Consequently, an appropriate particle size distribution can optimize the mechanical properties and microstructure of ceramic cores while maintaining slurry stability.

**Figure 3:**
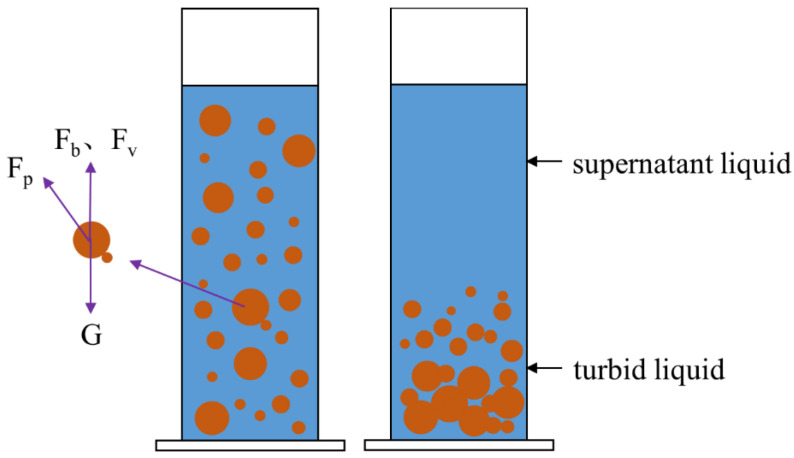
Settlement and force of ceramic particles in slurry.

The varying settling behaviors of particles in slurries are primarily related to the forces acting upon them within the suspension. SiO_2_ particles in slurries are subject to multiple forces, and the direction and magnitude of the resultant force dictate the movement state of the SiO_2_ particles, which manifest macroscopically as sedimentation in the slurry. Figure 3 illustrates the forces and sedimentation of ceramic particles in the slurry.

As depicted in Figure 3, the vertical forces acting on particles in the slurry include gravity (G), buoyancy (F_b_), viscous drag (F_v_) during particle movement, and the vertical component force (F_p_) of interactions between particles. The sedimentation of ceramic particles in the slurry is influenced by the forces acting upon them. As depicted in Figure 2a, slurries with a monomodal particle size distribution exhibit varying settling velocities among different particle sizes, leading to significant differences in the final sedimentation coefficients. The 1 μm particles show no obvious sedimentation, while particles with a diameter of 4 μm and above undergo significant sedimentation. According to Stokes’ formula, the settling velocity of particles in a slurry varies with particle size; within the same slurry, larger particles settle faster than smaller ones. When incorporating ceramic particles of different sizes, or adopting a multi-modal particle distribution, the settling velocity of the particles is altered by changing their radius, thereby modifying the force acting on the particles in the slurry. This change in settling velocity, influenced by the interaction among the ceramic particles, reduces the overall sedimentation velocity and, consequently, enhances the stability of the slurry.

**Figure 4:**
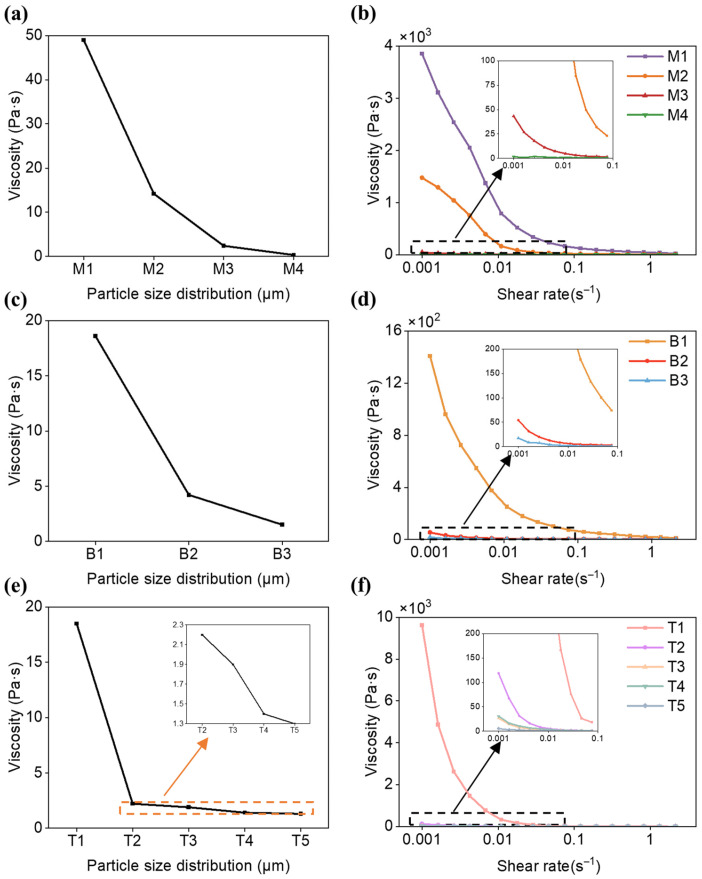
Printability of the SiO_2_ ceramic slurries with different particle size distributions. Viscosity: (**a**) monomodal particle size distributions; (**c**) bimodal particle size distributions; (**e**) trimodal particle size distributions. Rheological properties: (**b**) monomodal particle size distributions; (**d**) bimodal particle size distributions; (**f**) trimodal particle size distributions.

Figure 4 illustrates the trend of slurry viscosity as a function of particle size for various particle size distribution. Within the monomodal particle size distributions, the viscosity of the slurry significantly decreases with the increase in particle size. Within the same particle size distribution, the lower the proportion of smaller particles, the lower the slurry viscosity. Between different particle size distributions, the more diverse the particle sizes, the lower the slurry viscosity. Smaller particles, due to their higher specific surface area, enhance the interaction forces and frictional resistance between ceramic particles and the resin system [50]. Moreover, smaller particles are more prone to agglomeration, leading to an increase in slurry viscosity. Under identical solid loading conditions, slurries containing smaller ceramic particles have a higher particle count. An increase in the number of particles leads to a more pronounced impediment to internal relative motion within the slurry, consequently resulting in higher viscosity. Compared to alterations in particle size, variations in the proportion of different particle sizes have a relatively minor impact on the viscosity of the slurry.

Furthermore, the rheological properties of SiO_2_ ceramic slurries with varying particle size distributions were investigated. As observed in Figure 4, the slurry viscosity exhibited a significant decrease with the increase in shear rate, demonstrating the typical characteristics of a non-Newtonian fluid. The slurry within the reservoir exhibited a certain degree of fluidity, and with the assistance of a blade, the viscosity was reduced, allowing the slurry to be uniformly spread across the reservoir, thereby exhibiting printability.

Overall, compared with the unimodal and bimodal systems, the trimodal particle-size distribution exhibited a more favorable balance between slurry stability and rheological behavior for stereolithography processing. The unimodal system showed relatively poor suspension stability due to the easier sedimentation of larger particles. Although the bimodal system reduced the sedimentation coefficient, the trimodal system further improved slurry stability by decreasing the sedimentation coefficient to below 20% for appropriate particle-size combinations. In addition, the multimodal particle-size distribution maintained the slurry viscosity within a suitable range for recoating and printing. A lower slurry viscosity is beneficial for stereolithography because it improves the flowability and spreading behavior of the slurry during recoating, contributing to the formation of a more uniform coating layer and reducing printing defects such as pores, delamination, and uneven surfaces. Furthermore, lower viscosity facilitates the rearrangement and packing of ceramic particles, which is beneficial for curing accuracy and dimensional precision of the printed green bodies. Therefore, the trimodal system was considered the most suitable particle-size configuration for stereolithography processing.

### 3.2. Effects of Nanopowder

The research illustrates that particles of different sizes exhibit varying settling behaviors in slurries, which consequently impacts the slurry’s viscosity. Generally, particles with smaller diameters are less likely to settle within the slurry. However, particles with smaller sizes tend to result in higher viscosities, which poses challenges for the preparation of high solid loading slurries [48]. The aforementioned experiments indicate that ceramic particles with a diameter of 1 μm can remain free of significant sedimentation for up to 264 h. Within the slurry, interparticle forces are present, which, for smaller particles, not only do they settle less readily, but they also disperse uniformly throughout the slurry. Upon contact with other particles, these small particles exert forces that hinder the settlement of neighboring particles. Consequently, based on our research with the trimodal particle size distributions of 23 μm:8 μm:4 μm, we attempted to investigate the effect of adding a small amount of nanopowder with a diameter of 20 nm on the stability of the slurry. Nanopowder was added to the ceramic powder at an addition amount of 1 wt.%, and the particle size distributions are presented in Supplementary Table S2 of Supplementary Materials.

**Figure 5:**
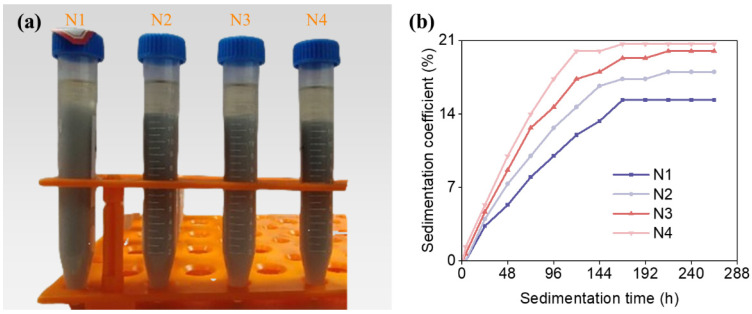
The change of delamination and sedimentation coefficient of adding nanopowder slurry with time after standing for 264 h: (**a**) delamination condition; (**b**) sedimentation coefficient.

Figure 5 illustrates the stratification of the slurry with nanopowder after 264 h of standing, as well as the change in the sedimentation coefficient over time. The results indicate that, in comparison to slurry without nanopowder, the slurry with nanopowder exhibits a lower sedimentation coefficient, a significantly slower sedimentation process, and an increased required settling time.

Compared with larger particles, the 20 nm SiO_2_ nanoparticles possess a much smaller particle size and are therefore less likely to sediment under the influence of gravity. Due to their extremely small size, Brownian-motion-like behavior may become increasingly important in the movement of the nanoparticles, allowing them to remain more uniformly dispersed within the slurry. Furthermore, the uniformly dispersed nanoparticles may partially hinder the sedimentation of larger particles, thereby reducing the overall sedimentation rate and sedimentation coefficient of the slurry. Therefore, the improved stability observed after nanopowder addition can be reasonably attributed to the combined effects of reduced particle size and enhanced particle dispersion.

**Figure 6:**
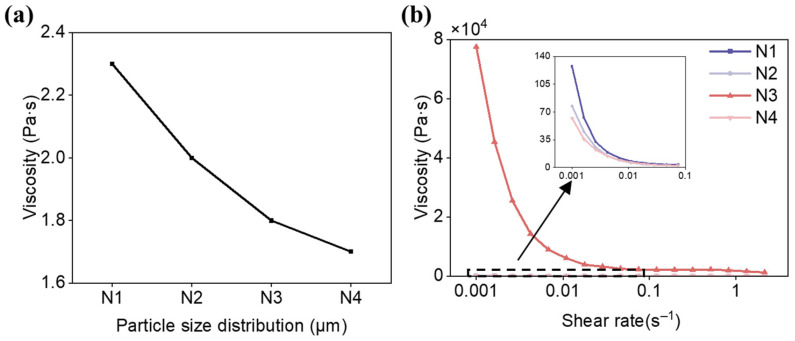
Printability of the SiO_2_ ceramic slurries with different particle size distributions added with nanopowder. (**a**) viscosity; (**b**) rheological properties.

The graph depicted in Figure 6 illustrates the relationship between the viscosity of a trimodal particle size distributions slurry with nanopowder. It is observed that as the proportion of larger particles increases, the viscosity of the slurry decreases. As the proportion of larger particles increases, the impact of adding nanopowder on the slurry’s viscosity becomes more pronounced, but the addition of a small amount of nanopowder to the 23 μm: 8 μm: 4 μm = 1:1:1 slurry has a minimal effect on its overall viscosity, maintaining it at a relatively low level. For instance, the slurry with the highest viscosity, which has a particle size distribution of 23 μm:8 μm:4 μm = 1:1:1, exhibits a viscosity of 2.3 Pa·s after the addition of nanopowder. This allows the slurry to spread evenly in the tank.

Figure 6b illustrates the rheological properties of the slurry after the addition of nanopowder. It is evident that the slurry’s viscosity decreases with the increase in shear rate, manifesting distinct non-Newtonian fluid characteristics. The incorporation of nanopowder does not alter the slurry’s rheological properties. When combined with Table 1, it is clear that the addition of a small amount of nanopowder does not impact the printability of the slurry with a particle size distribution of 23 μm: 8 μm: 4 μm = 1:1:1.

Due to the high specific surface area and strong interparticle interactions of nanopowder, as well as the frequent collisions caused by Brownian motion, the addition of nanopowder tends to increase the viscosity of the slurry to some extent. However, the small amount of nanopowder added, coupled with its fine particle size, allows it to fill the gaps between larger particles, resulting in a relatively minor impact on viscosity [50]. However, within different trimodal particle size distributions, the effect of nanopowder on increasing slurry viscosity becomes increasingly pronounced with the increase in the proportion of larger particles. This is because slurries with a higher proportion of larger particles have a relatively lower total number of particles. Consequently, when the same amount of smaller nanopowder particles is added, the nanopowder constitutes a relatively higher proportion in the slurry with a larger particle size, thereby exerting a more significant influence on the viscosity of the slurry. The sedimentation coefficients and viscosities of the suspensions with and without the addition of nanopowder for the trimodal particle size distributions are presented in Table 1.

**Figure 7:**
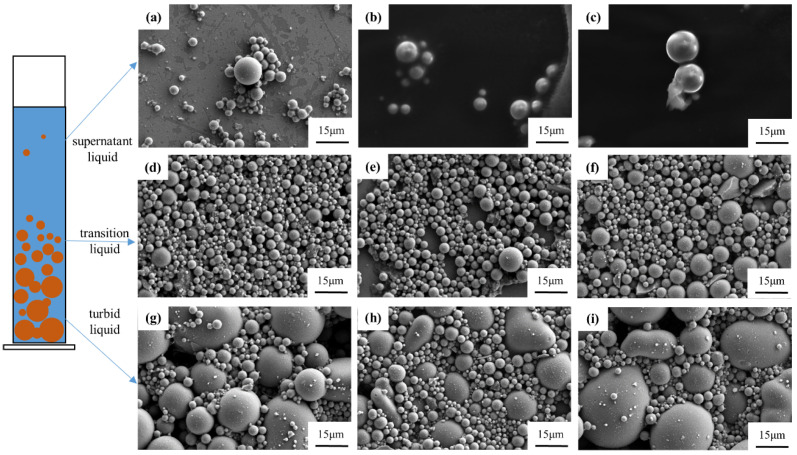
Particle distribution of each layer in different periods: (**a**) 96 h supernatant; (**b**) 192 h supernatant; (**c**) 144 h supernatant; (**d**) 96 h transition layer; (**e**) 144 h transition layer; (**f**) 192 h transition layer; (**g**) 96 h precipitation; (**h**) 144 h precipitation; (**i**) 192 h precipitation.

Take 1 mL slurry of the supernatant, the transition area between the supernatant and the sediment, and the turbid from the slurry at 96 h, 144 h, and 192 h, the rapid sedimentation period, slow sedimentation period, and stable period, respectively, to observe the particle size distribution in the slurry. Figure 7 illustrates that over time, from top to bottom within the test tube, the particle size in the slurry gradually increases. At 96 h of standing, the slurry is in the rapid sedimentation period, where the supernatant appears clear; however, after washing with anhydrous ethanol, fine particles can be observed within the sediment. These sediments are predominantly composed of particles of 3 μm and smaller, with a minor presence of particles around 5 μm. In the transition layer, the particle size distribution is relatively uniform, mainly consisting of particles around 5 μm, occasionally with particles exceeding 15 μm. In the turbid layer, an increased number of particles of 10 μm and larger are present, while the majority of particles remain around 5 μm. Additionally, after resting for 96 h, the particle sizes within each layer were relatively uniform.

By 144 h, the slurry enters the slow sedimentation period, where no significant particle sedimentation is observed after ethanol washing of the supernatant. The particle count is reduced, with a multi-modal particle size distribution predominantly below 10 μm. In the transition layer, most particles are around 3–5 μm, with a smaller portion reaching approximately 10 μm, and a few particles exceeding 10 μm. In the turbid layer, the number of particles larger than 10 μm increases.

At 192 h of resting, only a few particles around 10 μm are observed in the supernatant. The turbid layer exhibits a wider range of particle sizes, with most around 5 μm, but some reach approximately 15 μm. Compared to the 96 h and 144 h, there is a notable increase in the number of larger particles. In the turbid layer, in addition to some particles exceeding 15 μm, the majority of particles fall between 5–10 μm in size.

It is evident that as sedimentation time increases, particles settle, leading to a gradual reduction in the number of particles in the supernatant, while the number of particles in the transition and sedimentation layers gradually increases. Figure 7 reveals that during the early sedimentation phase, particles larger than 5 μm are primarily responsible for settling. Particles larger than 5 μm are mainly distributed in the transition and turbid layer, with larger particles settling at a faster rate. Consequently, the turbid layer exhibits larger particle sizes compared to the transition layer, although the particle size distribution within both layers remains relatively uniform, indicating ongoing sedimentation and a general downward movement of particles, with larger particles moving slightly faster. As sedimentation progresses, the number of particles in the supernatant decreases, leading to a reduction in slurry viscosity and the viscous force acting on the particles, which accelerates the settling rate. As a result, smaller particles also begin to settle during the post-sedimentation period. This phase is characterized by a sharp decrease in the number of particles in the supernatant, with only a few particles remaining, exhibiting an uneven size distribution. Particles size around 3 μm, initially present in the supernatant during the early sedimentation phase, are now predominantly found in the transition layer, leading to a gradient distribution of particle sizes, with a focus around 3–5 μm. Additionally, particles size around 15 μm start to appear in the turbid layer, indicating that a portion of the larger particles has completed sedimentation. By 192 h, the majority of particles in the supernatant have completed sedimentation. The transition layer now contains particles ranging from around 3 μm to 15 μm. In the turbid layer, there is an increased proportion of particles larger than 15 μm.

### 3.3. Effects of Solid Loading

**Figure 8:**
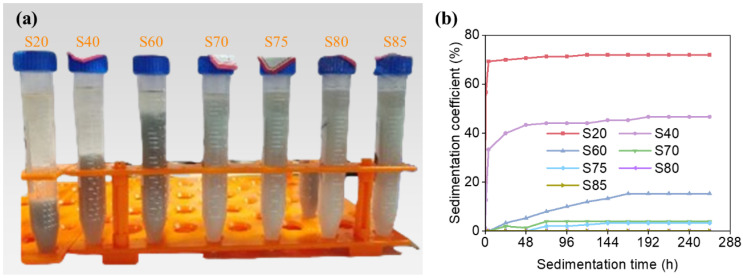
Changes of delamination and sedimentation coefficient of slurry with different solid loading after standing for 264 h: (**a**) delamination condition; (**b**) sedimentation coefficient.

The slurries with solid loadings 20 wt.%, 40 wt.%, 60 wt.%, 70 wt.%, 75 wt.%, 80 wt.%, 85 wt.% are correspondingly named as S20, S40, S60, S70, S75, S80, S85, respectively. Figure 8 illustrates the stratification behavior and the temporal evolution of the sedimentation coefficient for slurry samples with varying solid loading after a 264 h stable period. It is evident that as the solid loading increases, the sedimentation coefficient of the slurry gradually decreases, and the settling velocity slows down. With the increase in solid loading, the number of particles in the slurry increases, and they become more uniformly dispersed. This results in a reduction in the volume occupied by individual particles and the interparticle spacing, leading to more obstacles encountered during sedimentation. Consequently, at higher solid loading, particles experience greater hindrance during settling, resulting in smaller sedimentation coefficient and longer settling times.

For solid loadings of 20 wt.% and 40 wt.%, we observed a rapid sedimentation rate in the pre-sedimentation period, indicating that particle sedimentation was less hindered at this stage. When the solid loading exceeds 60 wt.%, the sedimentation coefficient of the slurry can be maintained at a low level. Specifically, when the solid loading exceeds 70 wt.%, the sedimentation coefficient can be reduced to less than 4%. When the solid loading reaches or exceeds 80 wt.%, there is no evident settling phenomenon, and the sedimentation coefficient is 0, indicating the successful preparation of a slurry with both high stability and high solid loading.

**Figure 9:**
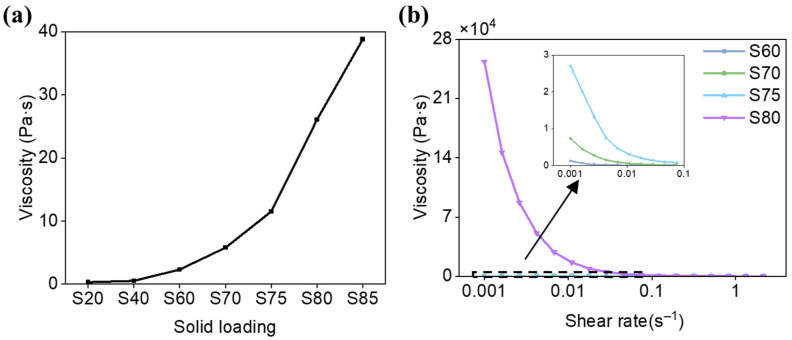
Printability of the SiO_2_ ceramic slurries with different solid loading: (**a**) viscosity; (**b**) rheological properties.

The variation in viscosity with solid loading is depicted in Figure 9. It is observed that as the solid loading increases, the viscosity of the ceramic slurry rises sharply. This phenomenon can be attributed to several factors. Firstly, the increase in solid loading leads to a higher particle concentration in the slurry, which intensifies the interactions between particles. When the interparticle spacing decreases, the forces of attraction between particles increase, making it more challenging for the particles to move. This, in turn, contributes to the increase in slurry viscosity. An analysis of the rheological properties of the slurry reveals that slurries with different solid loading also follow the typical non-Newtonian fluid behavior, with viscosity decreasing as the shear rate increases. In practical printing processes, with the assistance of a blade, the viscosity of the slurry is reduced, thus demonstrating its printability.

When the solid loading is below 60 wt.%, the viscosity of the slurry remains in a relatively low range. However, once the solid loading surpasses 70 wt.%, the viscosity of the slurry rises rapidly. It is noteworthy that a 5 wt.% increase in solid loading can lead to a 50% to 100% increase in slurry viscosity. This effect is particularly pronounced when the solid loading reaches 85 wt.%, at which point the viscosity of the slurry becomes too high for even distribution within the tank. Such high viscosity slurry is unsuitable for use as a printing slurry as it can impede the smooth progress of the printing process and compromise the final print quality. Therefore, although increasing the solid loading can further improve slurry stability, excessively high solid loading leads to poor recoating and spreading behavior due to the sharp increase in viscosity. In the present study, 80 wt.% was considered to be a suitable practical upper limit for stereolithography processing, providing a favorable balance between slurry stability and printability.

To ensure that the ceramic slurry is suitable for stereolithography processing, it is important to maintain the slurry viscosity within an appropriate range. However, slurry viscosity is not an independent parameter, but rather the combined result of multiple factors, including particle size, particle-size distribution, solid loading, and interparticle interactions. Therefore, viscosity can be regarded as a comprehensive rheological parameter that reflects the overall printability and flow behavior of the ceramic slurry.

In summary, through an analysis of the impact of particle size gradation, the addition of nano-powder, and the solid loading on the stability and viscosity of SiO_2_ ceramic slurry, the optimal parameters for photo-cured SiO_2_ slurry have been determined. The slurry utilizes spherical SiO_2_ powder with a trimodal particle size distribution of 23 μm: 8 μm: 4 μm at a ratio of 1:1:1. Additionally, 20 nm SiO_2_ powder is added to the slurry, with a mass fraction of 1 wt.% of the ceramic powder.

Stereolithography additive manufacturing technology was employed to fabricate intricate SiO_2_ ceramic green body. This ceramic green body was then subjected to a carefully designed sintering process, resulting in the production of silica-based ceramic cores with high dimensional accuracy, porosity, and mechanical properties. We have successfully fabricated ceramic cores green body with complex structures, as illustrated in Supplementary Figure S1 of Supplementary Materials. Our findings offer a theoretical basis and a practical approach for the preparation of highly stable and high solids loading slurries, and demonstrating the potential for long-term storage of slurries and the printing of large-sized ceramic green body in the stereolithography ceramic core fabrication process.

Higher slurry stability and more uniform particle dispersion are beneficial for stereolithography processing because they help suppress particle segregation and concentration gradients during long-duration printing. Improved slurry uniformity contributes to more homogeneous layer formation and particle packing in the green body, which is favorable for dimensional accuracy and sintering uniformity. In contrast, nonuniform particle distribution may lead to uneven thermal decomposition behavior and heterogeneous particle packing during sintering, thereby increasing the risk of crack formation, nonuniform pore distribution, and deterioration of mechanical properties. Therefore, slurry stability and dispersion not only influence the rheological behavior during printing, but also affect the microstructural uniformity and final properties of the printed ceramics.

Furthermore, shrinkage, porosity, and mechanical property measurements were conducted to verify the feasibility of fabricating ceramic components using the optimized slurry system. The slurry consisted of a particle-size distribution of 23 μm:8 μm:4 μm in a ratio of 1:1:1, with the addition of 1 wt.% 20 nm SiO_2_ nanopowder and a solid loading of 80 wt.%. The results showed that the shrinkage rate was maintained below 4%, the porosity was 21.56%, and the flexural strength reached 20.53 MPa.

## 4. Conclusions

Stereolithography additive manufacturing offers a feasible approach for the fabrication of silica-based ceramic cores. In this study, we systematically investigated the stability of SiO_2_ ceramic slurries. The effects of particle size distribution, nanopowder, and solids loading on the stability of SiO_2_ slurries were studied in detail. The key conclusions are as follows:

(1) The impact of particle size distributions on slurry stability and viscosity was examined. It was determined that smaller particle sizes lead to better slurry stability, with higher viscosities. A multi-modal particle size distribution can mitigate the settling of the slurry and maintain a lower viscosity level.
(2) The influence of nanopowder on slurry stability and viscosity was analyzed. The addition of 1 wt.% nanopowder significantly reduced the settling of the slurry, prolonged the settling process, and had minimal impact on the slurry viscosity.
(3) The particle size distribution of a slurry with a solid loading of 60 wt.%, particle size distribution of 23 μm:8 μm:4 μm = 1:1:1, and the addition of 1 wt.% nanopowder were investigated at various settling stages and within each settling layer.
(4) The effect of solid loading on slurry stability and viscosity was studied. When the solid loading is 80 wt.% or higher, the slurry can maintain prolonged stability without any evident settling. However, when the solid loading reaches 85 wt.% or higher, the slurry viscosity becomes too high for spreadability. The maximum solid loading for printing and maintaining stability over an extended period is 80 wt.%. Using the optimized slurry, ceramic samples with a shrinkage rate below 4%, a porosity of 21.56%, and a flexural strength of 20.53 MPa were successfully fabricated, demonstrating the feasibility of the developed slurry system for stereolithography processing.

In conclusion, a SiO_2_ slurry with high solid loading, suitable viscosity, and prolonged stability for stereolithography additive manufacturing was successfully developed, enabling the fabrication of silica-based ceramic components with good dimensional accuracy and mechanical properties. These findings provide useful guidance for the design and optimization of SiO_2_ ceramic slurries for stereolithography applications. Further validation involving large-scale ceramic core fabrication and extended continuous printing under practical processing conditions will be considered in future work.

## Figures and Tables

**Table 1:** The sedimentation coefficient and viscosity of the trimodal particle size without adding nanopowder and added nanopowder.

Particle Size Distribution	Sedimentation Coefficient (%)		Viscosity (Pa·s)	
	Without Nanopowder	Nanopowder	Without Nanopowder	Nanopowder
23 μm:8 μm:4 μm = 1:1:1	16.67	15.3	2.2	2.3
23 μm:8 μm:4 μm = 5:3:2	18.67	18.00	1.9	2.0
23 μm:8 μm:4 μm = 6:3:1	23.33	20.00	1.4	1.8
23 μm:8 μm:4 μm = 7:2:1	24.67	20.67	1.3	1.7

## Data Availability

The original contributions presented in this study are included in the article/Supplementary Materials. Further inquiries can be directed to the corresponding author.

## Supplementary Materials

The following supporting information can be downloaded at: https://www.mdpi.com/article/10.3390/ma19102071/s1. Table S1: Particle size distribution information of SiO2 slurries with monomodal, bimodal, and trimodal systems. Table S2: Particle size distribution information of trimodal slurries with na-nopowder addition. Figure S1: Photographs of stereolithography-fabricated SiO2 ceramic green bodies with complex structures.

## Abbreviations

The following abbreviations are used in this manuscript:

SL	Stereolithography
HDDA	1,6-hexadiol diacrylate
TMPTA	1,1,1-trimethylpropane triacrylate
TPO	diphenyl (2,4,6-trimethylbenzoyl) phosphine oxide
