# Supplementary material for: The High Solid Loading and Stability of SiO2 Ceramic Slurry for Stereolithography
Source: Materials (Basel). 2026 May 15;19(10):2071. doi: 10.3390/ma19102071 (PMC13208947; doi:10.3390/ma19102071)
Supplement: Supplementary file 1 [file materials-19-02071-s001.zip › materials-4307227-supplementary.pdf]

Supplementary Table S1

Particle size distributions of slurry

Particle size distributions	Particle size	Distributions	Table
Monomodal particle size distributions	1μm	1	M1
	4μm	1	M2
	8μm	1	M3
	23μm	1	M4
Bimodal particle size distributions	4μm:1μm	1:1	B1
	8μm:4μm	1:1	B2
	23μm:8μm	1:1	B3
Trimodal particle size distributions	8μm:4μm:1μm	1:1:1	T1
	23μm:8μm:4μm	1:1:1	T2
	23μm:8μm:4μm	5:3:2	T3
	23μm:8μm:4μm	6:3:1	T4
	23μm:8μm:4μm	7:2:1	T5

Supplementary Table S2

Particle size distributions of slurry

Particle size	Distributions	Nanopowder（wt%）	Table
23μm:8μm:4μm	1:1:1	1	N1
23μm:8μm:4μm	5:3:2	1	N2
23μm:8μm:4μm	6:3:1	1	N3
23μm:8μm:4μm	7:2:1	1	N4

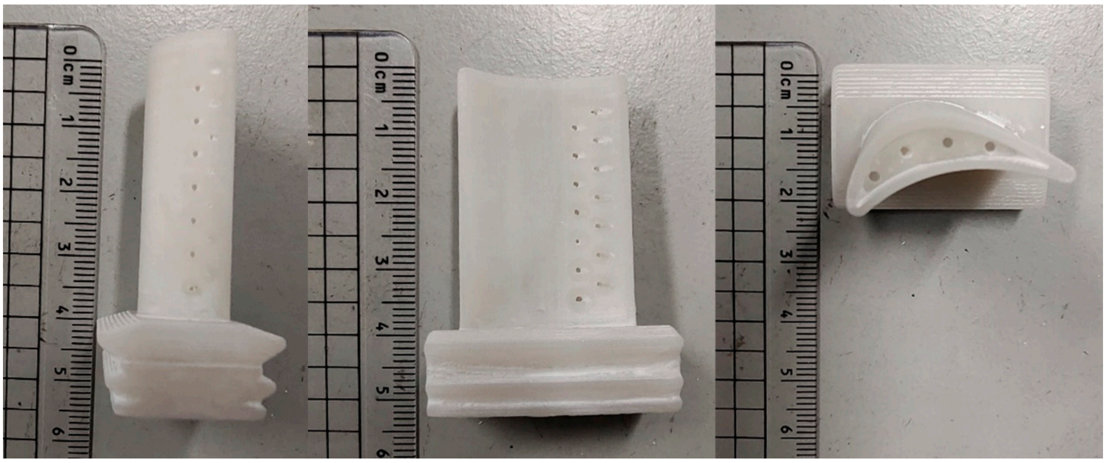

Supplementary Figure S1. Complex shape ceramic core billet and sintered part prepared by DLP
